# Explainable machine learning prediction of internet addiction among Chinese primary and middle school children and adolescents: a longitudinal study based on positive youth development data (2019–2022)

**DOI:** 10.3389/fpubh.2025.1590689

**Published:** 2025-07-16

**Authors:** Jiahe Liu, Lang Chen, Yuxin Chen, Jingsong Luo, Kexin Yu, Linlin Fan, Chan Yong, Huiyu He, Simei Liao, Zongyuan Ge, Lihua Jiang

**Affiliations:** ^1^AIM for Health Lab, Monash University, Melbourne, VIC, Australia; ^2^School of Mathematics and Statistics, University of Melbourne, Melbourne, VIC, Australia; ^3^Jockey Club School of Public Health and Primary Care, The Chinese University of Hong Kong, Hong Kong, Hong Kong SAR, China; ^4^West China School of Public Health and West China Fourth Hospital, Sichuan University, Chengdu, China; ^5^School of Nursing, Capital Medical University, Peking, China; ^6^General Practice Ward, International Medical Center Ward, General Practice Medical Center, West China Hospital, Sichuan University, Chengdu, China

**Keywords:** internet addiction, adolescent and children, machine learning, extra random forest, longitudinal study

## Abstract

**Background:**

Internet Addiction (IA) has emerged as a critical concern, especially among school age children and adolescents, potentially stalling their physical and mental development. Our study aimed to examine the risk factors associated with IA among Chinese children and adolescents and leverage explainable machine learning (ML) algorithms to predict IA status at the time of assessment, based on Young’s Internet Addiction Test.

**Methods:**

The longitudinal data consisting of 8,824 schoolchildren from the Chengdu Positive Child Development (CPCD) survey were analyzed, where 33.3% of participants were identified with IA (Age: 10.97 ± 2.31, Male: 51.73%). IA was defined using Young’s Internet Addiction Test (IAT ≥ 40). Demographic variables such as age, gender, and grade level, along with key variables including scores of Cognitive Behavioral Competencies (CBC), Prosocial Attributes (PA), Positive Identity (PI), General Positive Youth Development Qualities (GPYDQ), Life Satisfaction (LS), Delinquent Behavior (DB), Non-Suicidal Self-Injury (NSSI), Depression (DP), Anxiety (AX), Family Function Disorders (FF), Egocentrism (EG), Empathy (EP), Academic Intrinsic Value (IV), and Academic Utility Value (UV) were examined. Chi-square and Mann–Whitney U tests were employed to validate the significance of the mentioned predictors of IA. We applied six ML models: Extra Random Forest, XGBoost, Logistic Regression, Bernoulli Naïve Bayes, Multi-Layer Perceptron (MLP), and Transformer Encoder. Performance was evaluated via 10-fold cross-validation and held-out test sets across survey waves. Feature selection and SHapley Additive exPlanations (SHAP) analysis were utilised for model improvement and interpretability, respectively.

**Results:**

ExtraRFC achieved the best performance (Test AUC = 0.854, Accuracy = 0.798, F1 = 0.659), outperforming all other models across most metrics and external validations. Key predictors included grade level, delinquent behavior, anxiety, family function, and depression scores. SHAP analysis revealed consistent and interpretable feature contributions across individuals.

**Conclusion:**

Depression, anxiety, and family dynamics are significant factors influencing IA in children. The Extra Random Forest model proves most effective in predicting IA, emphasising the importance of addressing these factors to promote healthy digital habits in children. This study presents an effective SHAP-based explainable ML framework for IA prediction in children and adolescents.

## 1 Background

The proliferation of the internet has significantly increased the vulnerability of children to internet addiction (IA), as they are particularly susceptible due to their evolving cognitive functions (1, 2). IA has been acknowledged in medical literature as “Internet Gaming Disorder” by the Diagnostic and Statistical Manual of Mental Disorders (DSM-5) (3) and “Gaming Disorder” by the International Classification of Diseases (ICD) (4) with a focus on specific online activities that can become addictive (5).

IA’s prevalence among children has drawn increasing concerns due to its serious developmental and psychological consequences. As children have underdeveloped self-regulation and cognitive control, they are more vulnerable toward IA (6). This vulnerability may lead to consequences such as compulsive use, withdrawal signs, and tolerance, often linked with sleep issues, attention deficits, depression, anxiety, and even suicidality (2, 7–10).

On the other hand, the risk of IA in children is influenced by an interplay of sociodemographic, psychological, and familial factors (6). For sociodemographic factors such as age, gender, and socioeconomic status, studies have shown that males are more likely to develop IA compared to females, possibly due to a higher tendency to engage in gaming and online risk-taking behaviors (11–13). Additionally, younger adolescents with limited impulse control are more susceptible (14), and children from lower socioeconomic backgrounds may face fewer parental restrictions and greater emotional vulnerability (15). For psychological factors, including mental health variables such as depression, anxiety, loneliness, low self-esteem, and impulsivity, studies suggest that impulsivity and poor self-regulation play a particularly crucial role in the development and severity of IA. For instance, Fan et al. (16) showed that adolescents who experienced childhood trauma are at significantly higher risk of IA, likely due to emotional dysregulation. Similarly, Jeong et al. (17) found that poor self-control and emotional vulnerability predicted the persistence of internet gaming disorder over time. For familial factors, which relate to parenting style, parental monitoring, and the quality of parent–child relationships, multiple studies point to the importance of warm, consistent, and involved parenting. Lee and Kim (18) found that IA in children is significantly associated with both parental characteristics (e.g., low emotional warmth, inconsistent discipline) and lack of parental monitoring, underscoring the protective role of engaged caregiving. Similarly, Karaer et al. (19) found that parents of adolescents with IA often exhibited lower levels of acceptance, monitoring, and emotional availability. In addition, Koca et al. (20) highlight how unregulated internet use patterns can also co-occur with other behavioral vulnerabilities, such as food addiction, in children. These domains collectively informed our selection of variables from the Positive Youth Development (PYD) dataset, though we acknowledge that our study does not aim to provide an exhaustive list of IA predictors. Rather, we grounded our choices in empirical literature and theoretical models, especially those focusing on developmental vulnerabilities and socio-ecological frameworks of risk.

The serious impacts and complicated risk factors of IA in children necessitate early intervention and prevention, highlighting the need for incorporating Machine Learning (ML) approaches. Implementing ML can automate the analysis typically done by experts (21, 22). By leveraging historical data and statistical inference, ML significantly enhances the identification of patterns in data, facilitating tasks such as clustering, classification, and prediction (23, 24). In the context of ML, key aspects include predicting the value or category of a variable, and improving the model’s performance and its generalisability (25). ML is crucial in public health for identifying at-risk populations for adverse health outcomes and developing targeted interventions (26).

Research has established ML’s utility in identifying IA, linking psychological, physiological, and behavioral indicators to addiction patterns. ML has been paired with neuroscience, notably in analysing fMRI images to track brain changes related to IA, thereby informing predictive models (27). Studies measuring traits including anxiety, depression, ADHD, impulsiveness, obesity, and personality have utilised ML in predicting IA symptoms (28–31). Web usage patterns and the Covid19 pandemic’s impact have also been analyzed through ML, revealing behavioral links to IA (32, 33).

However, existing ML-based studies on IA primarily focus on improving predictive performance, with limited attention to model interpretability (34–36). As a result, it remains unclear how specific features influence IA risk, hindering practical application in prevention and intervention efforts. To address this gap, our study integrates SHapley Additive exPlanations (SHAP) (37), enabling not only accurate prediction but also a clearer understanding of the direction and magnitude of each predictor’s influence on IA outcomes.

Moreover, IA studies often overlook younger demographics, focusing mainly on college students, which may skew data representation (27, 28, 30, 31, 33). Small sample sizes in such research further challenge the generalisability of findings (31). These issues emphasize the need for broader ML application in IA research. Therefore, this study aims to refine ML algorithms using longitudinal data, identifying the most accurate and interpretable model for predicting IA risk among Chinese primary and middle school children and adolescents (Grades 1 to 9).

## 2 Methods

### 2.1 Study design

The participants of this study were primary and middle school students (Grade 1–9) and their parents from 5 different schools through a cluster sampling strategy, covering both urban districts and suburban regions of Chengdu. The schools included a mix of primary and middle schools serving students from Grade 1 to Grade 9, and varied in socioeconomic and geographic profiles, which helps improve within-region representativeness. This study was a dynamic cohort study, wave one of surveys was concluded between 23 December 2019 and 13 January 2020, while three follow-up surveys were collected approximately every 12 months later. Students were recruited as full cohorts from selected grades within each school, as detailed in the CPCD cohort protocol. However, students who graduated from primary or middle school during the follow-up period were not tracked to their new schools. The data were collected using questionnaires from the CPCD survey, which were previously published by Zhao et al. (38). Participants completed the questionnaires independently in classrooms under the supervision of two well-trained research assistants. Participants tend to take an average of 10 min to complete the questionnaire, which are immediately returned to the researchers upon completion. This research was conducted with strict compliance to the Helsinki Declaration’s principles, and the data was anonymized so to protect individual privacy during research output publication. This cohort study was approved by the Medical Ethics Committee of Sichuan University (Grant No. K2020025) and written informed consent from each students’ legal guardian is available. Further details on this study could be found in the published cohort (38).

In total, 12,977 students undertook the survey at least once during its four waves as part of this study according to the distinct unique identifier count in the database. Our study is aimed to evaluate associations between positive youth development and IA among children and adolescents (Grades 1 to 9). For modelling purposes of temporal order verification (i.e., model parameter fitting, hyperparameter tuning, and model selection), only the data of 8,824 students who participated in wave 1 were used, while the remaining waves were made into 3 separate out-of-sample distinct datasets (wave 2 with 7,936 participants, wave 3 with 8,250 participants, and wave 4 with 5,113 participants). Due to COVID-19-related disruptions in school schedules and survey administration, especially in Waves 2 to 4, the number of participants decreased compared to Wave 1. Also, we divided the participants into different groups according to students’ belonged schools for external verification. This opened the valuable opportunity to observe model performance on out-of-sample data from a different temporal segment as well samples from a completely different time and underlying group of participants, to further validate the model.

### 2.2 Internet addiction definition and measurement

This study adopted Young’s definition of IA, viewing it as an inability to control impulses without the influence of external substances. The Young Internet Addiction Test (IAT) was used to measure whether IA occurrence. The items are summed to obtain a total score using the 0 (rarely or none of the time) to 5 (most or all of the time) scores for individual items and A higher score reflects greater symptoms of IA, the IAT score ≥ 40 is typically employed as a cut-off for clinical IA (10).

### 2.3 Predictors selection and measurement

Our study included 19 predictive variables, guided by a review of the existing literature and the foundational architecture of our model. This selection process involved the direct measurement of demographic variables, including age, gender, and grade level, alongside a collection of variables aimed at evaluating the psycho-social development of youth. Height and weight were included as standard physical health indicators in youth surveys and may serve as proxy measures for general well-being and potential obesity, which has been linked in prior studies to problematic internet use and adverse mental health outcomes (39). Including such indicators allows for a more comprehensive assessment and the possibility of detecting indirect effects. These evaluations utilized a variety of scales, as implemented in the CPCD survey by Zhao et al. (38). For the psycho-social variables, four key Positive Child Development (PCD) traits including Cognitive Behavioral Competencies (CBC), Prosocial Attributes (PA), Positive Identity (PI), and General Positive Youth Development Qualities (GPYDQ) were measured by the Chinese Positive Youth Development Scale (40). Life Satisfaction (LS) was measured by the satisfaction with life scale (41). Delinquent Behavior (DB) was assessed through 12 questions regarding the frequency of students’ engagement in various misbehavior throughout the past year (42). Non-Suicidal Self-Injury (NSSI) was measured by the Deliberate Self-Harm Inventory (43). Depression (DP) was measured by the Center for Epidemiological Studies-Depression Scale (44), Anxiety (AX) was measured by Screen for Child Anxiety Related Disorders (45), Family Function (FF) Disorders was measured by the Chinese Family Assessment Instrument (46) Egocentrism (EG) was measured by the Chinese Adolescent Egocentrism Scale (47), alongside Empathy (EP), and Academic Intrinsic Value (IV) and Academic Utility Value (UV) were measured by specifically tailored questions for the CPCD cohort (48). Except for age, demographic information was categorized as categorical data within our ML prediction model. Supplementary Table A1 offers an extensive overview of the tools and scales utilized for these measurements.

### 2.4 Statistical analysis

The study conducted descriptive statistical analyses, calculating mean and variance, as well as examining the distribution of all demographic variables, positive child development indicators, and IA variables included in the research. Spearman’s correlation test was used to assess co-linearity among predictive features, with only one feature in each significantly correlated pair (*r* > 0.8) retained. The Mann–Whitney U test and Chi-square test was conducted to assess whether the distribution of a particular predictive feature (continuous and categorical, respectively) was significantly different for individuals with and without IA symptoms. All statistical analyses were conducted using SPSS software (Version 29.0.2.0 (20), IBM, Inc., Chicago, IL).

### 2.5 Machine learning

#### 2.5.1 Data pre-processing

The phenomenon of missing values in certain columns was observed for several rows in the dataset, and these rows were removed as such occurrences were infrequent and the remaining dataset size was sufficient for modelling purposes for this study. Categorical variables gender and grade were one-hot-encoded as demonstrated in Equation (1), while an alternative continuous encoding method was applied to grade as it had ordinal properties.


(1) $$f(x)=i-\frac{0.5}{m}$$

where *m* is the total number of classes, and *i* is the position of *x* in the ordinal variable, starting from 1.

The dataset was then split into model development (70%) and testing (30%) sets, stratified according to the target variable “IAT outcome,” along with School and Grade. Z-score normalisation was also separately applied on continuous feature columns for each of the model development sets’ 10-fold crossfold-validation (CV) training sets, with the training set fitted z-score parameters applied onto the corresponding model development sets’ 10-fold CV validation sets. Experiments were conducted for both where datasets used and did not use feature z-score, with the former outperforming the latter in terms of mean CV validation accuracy. As the labels in this dataset were imbalanced, with approximately 33% positive and 67% negative cases, up-sampling and down-sampling were experimented as class balancing techniques on each of the model development sets’ 10-fold CV training sets but produced inferior accuracy on the mean CV validation set compared to training on an imbalanced dataset. In the design of this experiment, we took great care to avoid data leakage from any of the sets whose results are used to evaluate or select models into the set employed to fit models.

#### 2.5.2 Model selection

This study selected six supervised machine learning algorithms to predict IA in our chort based on features derived from data collected in the surveys. The selected classifier algorithms are: Extra Random Forest Classifier (ExtraRFC), Bernoulli Naïve Bayes (BernoulliNB), Logistic Regression (LogisticReg), eXtreme Gradient Boosting Classifier (XGB), Multiple Layer Perceptron (MLP) and Transformer Encoder Classifier (Transformer). During experiments, 15 other common machine learning models such as ADABoost and LightGBM were trialled but their optimum 10-CV validation performance did not yield top-6, and hence will be abstained from discussion. ExtraRFC and XGB are ensemble learners built upon classification tree models – utilising the predictions from a large group of weak tree learners to make a robust final prediction. Specifically, each tree in the ExtraRFC model learns to predict the target, while in XGB each tree predicts the residual of the aggregated predictions from the previously fitted trees; both models use different randomly chosen sets of features and instances when fitting each tree. Bernoulli Naïve Bayes makes classification decisions based on the Bayes rule – taking the assumption that features are independent and that features follow the Bernoulli distribution conditioned to the class label (the model infers binarisation of continuous variables during training). LogisticReg classifies via a linear boundary separating the two classes in the space of the predictor variables, which is fitted by linearly regressing the log-odds of the target variable (0, 1). MLP and Transformers are deep learning models which consist of layers of neurons which performs non-linear transformation of input values into output values, which are then fed into the next layer as inputs; model parameters are fitted by back-propagation based on gradients derived from a loss function between predicted values and ground truth labels. Both are nonlinear models and universal approximators due to their ability to asymptotically perform like any functions given enough model depth (number of layers and number of neurons per layer). The transformer model architecture used in this study is similar to that of the BERT model designed for natural language processing problems, which only utilises the transformer encoder rather than the original encoder-decoder architecture. Transformers gain their performance mainly from the attention mechanism within their architecture.

#### 2.5.3 Model hyperparameter tuning and evaluation

10-fold CV was used to fit models and tune for optimal hyperparameters. The 10 training and validation folds were split from the model development set (70% of data), with none of the CV validation sets overlapping in instances (i.e., containing 7% of the data and CV training set containing 63% of the data - process illustrated in Supplementary Figure A4). The CV datasets were also split in a stratified manner as per school, grade, and IAT outcome. For each algorithm, the hyperparameter combination that gave the highest mean CV validation set accuracy score over all 10 sets was determined to be the best for building models on this data problem on this algorithm (optimal hyperparameters presented in Supplementary Table A2).

In this work, we have also included the number of features used as a tune-able hyperparameter, where hyperparameter values are: {most important feature}, {first two most important features}, {all features}. The importance of features was derived by first fitting an XGB model with default hyperparameters on each of the 10 CV training datasets before summing the feature importance values from each of these 10 models. This method has the advantage over using F-test and other feature selection methodologies in that it allows for considerations of interactions between variables in feature selection through using the XGB model to derive feature importance, while also allowing for the number of features ultimately used to be optimized based on the averaged CV validation dataset accuracy, thus avoiding the need of human arbitration. Constraints were placed on creating the sets of features which are hyperparameter values for the new hyperparameter “feature” in that if a one-hot-encoded variable (i.e., Grade 1) was included, then all other one-hot-encoded variables originally from the same feature must be included (i.e., Grade 2 – Grade 9). Local-greedy hyperparameter tuning strategies were employed to reduce the total number of combinations tuned for each model compared to grid search.

Two metrics used to quantitatively analyze model performance were the area under curve values for the receiver operator curve (AUC-ROC) and precision-recall curve (AP). The Delong test tests for significance in the difference between AUC-ROC of pairs of models, while the decision curve analysis (DCA) and calibration plot, respectively, calculate the net benefit at different probability thresholds and quantify the deviance of model predicted probabilities to ground truth. Common machine learning model metrics of accuracy, F1-score, precision, sensitivity, specificity, and negative predictive value (NPV) were also used to evaluate model performance as demonstrated in Equations (2–7). We chose the model with the general best validation metrics performance as the best model for this problem and evaluated its test set (illustrated in Supplementary Figure A4).


(2) $$\text{Accuracy}=\frac{\mathrm{TP}+\mathrm{TN}}{\mathrm{TP}+\mathrm{TN}+\mathrm{FP}+\mathrm{FN}}$$

(3) $$F1\;\text{Score}=\frac{2\times \text{precision}\times \text{recall}}{\text{precision}+\text{recall}}$$

(4) $$\text{Positive Predictive Value}=\frac{\mathrm{TP}}{\mathrm{TP}+\mathrm{FP}}$$

(5) $$\text{Sensitivity}=\frac{\mathrm{TP}}{\mathrm{TP}+\mathrm{FN}}$$

(6) $$\text{Specificity}=\frac{\mathrm{TN}}{\mathrm{TN}+\mathrm{FP}}$$

(7) $$\text{Negative Predictive Value}=\frac{\mathrm{TN}}{\mathrm{TN}+\mathrm{FN}}$$

where *TP* represents true positives, *TN* represents true negatives, *FP* represents false positives, and *FN* represents false negatives.

#### 2.5.4 Model interpretation

Machine learning algorithms may outperform traditional statistical algorithms for classification tasks in terms of predictive performance with their relaxation of distributional assumptions and better inductive bias that captures sharper classification signals. However, there is a trade-off for better predictive performance with model interpretability, and hence we use the SHapley Additive exPlanations (SHAP) algorithm to explain how models use the features to make predictions, which can serve as a proxy for understanding how these features interplay with the IAT outcome. SHAP is based on cooperative game theory and is applicable for any models with the advantages of efficiency where Shapley values sum up to the discrepancy between a prediction and the average predicted value, boasting symmetry, additivity, and consistency among its advantages. It can estimate the contributions to the prediction for each instance, where positive SHAP values indicate contributions toward classifying the instance as positive, while negative SHAP values indicate contributions toward classifying the instance as negative. All experiments were performed using Python version 3.9.18.

## 3 Results

### 3.1 Descriptive and inferential analysis

Among the 8,824 student responses collected in wave 1, 51.7% were male (*n* = 4,565), and 48.3% were female (*n* = 4,259), with education level ranging from Grade 1 to 9. In the first wave, 2,852 (33.3%) patients had positive IA diagnosis while 5,972 (67.7%) had negative.

As demonstrated in Table 1, the Mann–Whitney U and Chi-square test revealed that demographic factors including age, gender, grade, weight, height, along other social and psychological factors are significantly linked to IA.

**Table 1:** Chi-square analysis of IA risk factors.

Variables	Without IA (*n* = 5,972)	With IA (*n* = 2,852)	*P*-value
	Mean ± SD or *n* (%)	Mean ± SD or *n* (%)	
Age (years)	10.48 ± 2.16	11.99 ± 2.28	<0.001^a^
Gender			<0.001^b^
Male	2,935 (33.26%)	1,630 (18.47%)	
Female	3,037 (34.41%)	1,222 (13.85%)	
Grade			<0.001^b^
Grade = 1	343 (3.89%)	69 (0.78%)	
Grade = 2	316 (3.58%)	108 (1.22%)	
Grade = 3	922 (10.45%)	194 (2.20%)	
Grade = 4	924 (10.47%)	222 (2.52%)	
Grade = 5	962 (10.90%)	220 (2.49%)	
Grade = 6	894 (10.13%)	359 (4.07%)	
Grade = 7	704 (7.98%)	425 (4.82%)	
Grade = 8	486 (5.51%)	611 (6.92%)	
Grade = 9	421 (4.77%)	644 (7.30%)	
Weight (kg)	36.31 ± 12.86	42.93 ± 14.03	<0.001^a^
Height (cm)	140.59 ± 14.12	148.46 ± 14.23	<0.001^a^
CBC score	5.15 ± 0.82	4.62 ± 0.90	<0.001^a^
PA score	5.12 ± 0.91	4.63 ± 0.99	<0.001^a^
PIT score	5.01 ± 0.87	4.42 ± 0.97	<0.001^a^
GPYDQ score	5.09 ± 0.79	4.52 ± 0.89	<0.001^a^
LS score	4.60 ± 1.04	3.94 ± 1.17	<0.001^a^
DB score	0.16 ± 0.25	0.52 ± 0.74	<0.001^a^
NIS score	0.63 ± 1.99	2.59 ± 4.63	<0.001^a^
DP score	12.23 ± 8.75	19.25 ± 11.48	<0.001^a^
AX score	14.04 ± 12.81	23.63 ± 16.65	<0.001^a^
FF score	1.77 ± 0.66	2.27 ± 0.76	<0.001^a^
EG score	2.83 ± 0.88	3.19 ± 0.83	<0.001^a^
EP score	4.70 ± 0.88	4.23 ± 0.84	<0.001^a^
IV score	4.11 ± 0.75	3.48 ± 0.83	<0.001^a^
US score	4.48 ± 0.66	4.01 ± 0.79	<0.001^a^

^a^Mann–Whitney U test; ^b^Chi square analysis.

### 3.2 Prediction performance of different ML models for IAT outcome

**Figure 1:**
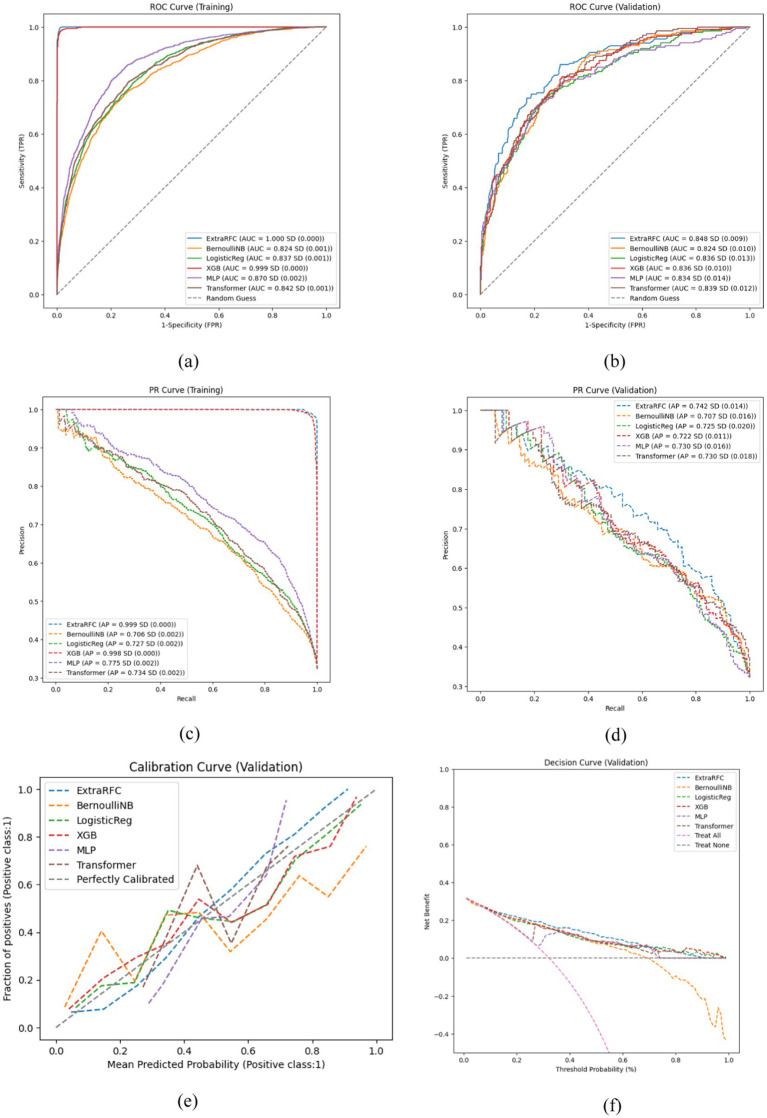
Key performance evaluation plots of 6 models. **(a)** ROC curve on the training set. **(b)** ROC curve on the validation set. **(c)** PR curve on the training set. **(d)** PR curve on the validation set. **(e)** Calibration curve on the validation set. **(f)** Decision curve on the validation set.

Figure 1 compares the ROC curves and PR curves of the different ML models predicting IAT outcome in children and adolescents (Grades 1 to 9) in both the training and validation sets. Out of the 6 models, ExtraRandomForest demonstrates the highest training and validation AUC value (AUC_T_ = 1.000; AUC_V_ = 0.848), AP value (AP_T_ = 0.999; AP_V_ = 0.743), accuracy score (Accuracy_T_ = 0.994; Accuracy_V_ = 0.795) and PPV value (AP_T_ = 0.998; AP_V_ = 0.730) out of all models. Additionally, ExtraRFC has the highest validation F1-score (F1_V_ = 0.650), equal first training F1 (F1_T_ = 0.974) tied with XGBoost and highest training specificity (Specificity_T_ = 0.999). The model with the highest training sensitivity is XGBoost (Sensitivity_T_ = 0.956) with ExtraRFC following closely at (Sensitivity_T_ = 0.951), while validation sensitivity is topped by BernoulliNB (Sensitivity_V_ = 0.651) with ExtraRFC ranking third at (Sensitivity_V_ = 0.578). For NPV score, once again ExtraRFC (NPV_T_ = 0.977) closely follows XGBoost (NPV_T_ = 0.979) for training, while in the validation set it (NPV_V_ = 0.817) is also a close second to BernoulliNB (NPV_V_ = 0.832). For specificity in the validation set, ExtraRFC (NPV_V_ = 0.898) is a close second to LogisticReg (NPV_V_ = 0.901). All scores for each model are provided in Table 2. The decision curve and calibration curve comparisons for each of the six models can be found in Figure 1. The DCA curve comparison suggest that all models except BernoulliNB have similar net benefits to each other in the validation set, with ExtraRFC maintaining the highest benefit for a large proportion of the threshold values (between 0.1 and 0.7) and delivering positive net benefit regardless of the threshold. ExtraRFC also tracks the real event perfect calibration curves most closely, with the least degree of deviation over all mean predicted probability values. The Delong test comparing AUC of pairs of models demonstrated ExtraRFC to be statistically significantly better performing at the task of predicting IAT outcome in children and adolescents (Grades 1 to 9) than all the other 5 models and *p*-values of each pair are presented in Supplementary Table A4. Overall, ExtraRFC dominated in almost most metrics and measures, especially in the out-of-sample validation set, and hence is the most well-rounded model for the task of explaining IAT outcome in children and adolescents (Grades 1 to 9).

**Table 2:** Model performance metrics of 6 models on 10-CV training and validation datasets.

Model	Sets	AUC	Accuracy	Sensitivity	Specificity	PPV	NPV	F1-score	AP
ExtraRFC	Training Validation	1.000 0.848	0.984 0.795	0.951 0.578	0.999 0.898	0.998 0.730	0.977 0.817	0.974 0.650	0.999 0.743
BernoulliNB	Training Validation	0.824 0.824	0.770 0.771	0.649 0.651	0.828 0.829	0.643 0.645	0.832 0.832	0.646 0.648	0.706 0.707
LogisticReg	Training Validation	0.837 0.836	0.786 0.786	0.545 0.546	0.901 0.901	0.724 0.725	0.806 0.806	0.622 0.623	0.727 0.725
XGBoost	Training Validation	0.999 0.836	0.983 0.783	0.956 0.583	0.997 0.879	0.993 0.698	0.979 0.815	0.974 0.635	0.998 0.722
MLP	Training Validation	0.871 0.834	0.803 0.780	0.586 0.551	0.906 0.889	0.749 0.705	0.821 0.806	0.657 0.618	0.775 0.730
Transformer	Training Validation	0.842 0.839	0.791 0.783	0.587 0.573	0.888 0.884	0.718 0.704	0.819 0.812	0.644 0.630	0.734 0.730

**Figure 2:**
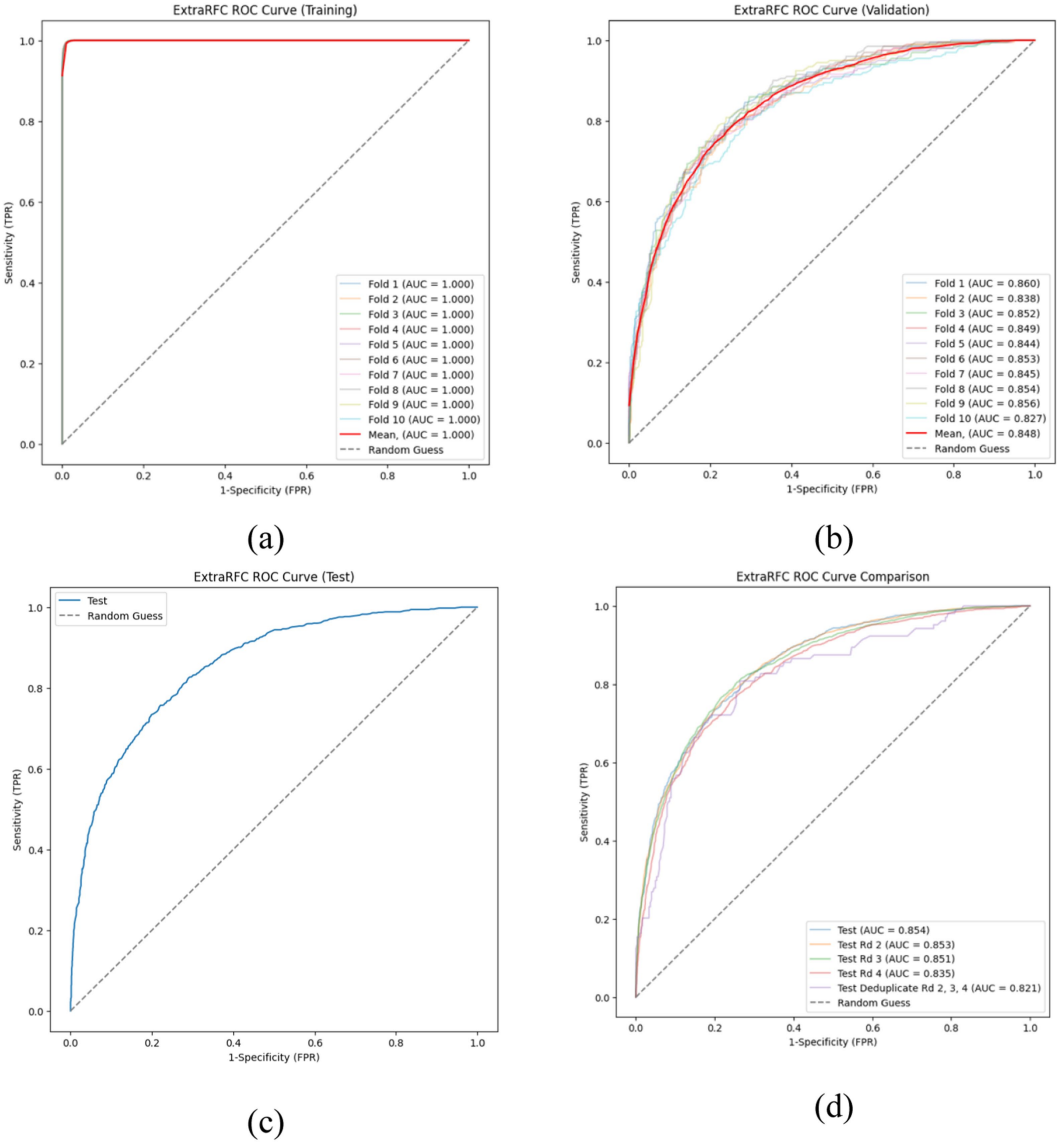
ROC curves of the ExtraRFC model across different data splits. **(a)** ROC curves on the training set across 10 folds. **(b)** ROC curves on the validation set across 10 folds. **(c)** ROC curve on the held-out test set. **(d)** ROC curve comparison across different test subsets.

Figure 2 presents ExtraRFC’s ROC plots for the training, validation (both 10-fold) and testing set(s) (including testing sets from other survey waves), and Table 3 details the key evaluation metrics for the three sets. The model performance for wave 2–4 as well as non-wave 1 test sets perform similarly well to the wave 1 testing set, albeit with slight decay in the non-wave 1 test set which was expected with both temporal and participant varied. The 10-fold training and validation PR plot, test (wave 1) DCA curve and calibration plot of ExtraRFC is supplied in Supplementary Figures A2, A3, respectively.

**Table 3:** Model performance metrics of ExtraRFC.

Sets	AUC	Accuracy	Sensitivity	Specificity	PPV	NPV	F1-Score
Train (10CV)	1.000	0.984	0.951	0.999	0.998	0.977	0.974
Validation (10CV)	0.848	0.795	0.578	0.898	0.730	0.817	0.650
Train (70%)	1.000	0.983	0.950	0.998	0.996	0.977	0.973
Test	0.854	0.798	0.605	0.890	0.724	0.825	0.659
Test (Rd 2)	0.853	0.798	0.543	0.915	0.745	0.814	0.628
Test (Rd 3)	0.851	0.800	0.547	0.912	0.800	0.820	0.626
Test (Rd 4)	0.836	0.819	0.473	0.933	0.701	0.843	0.565
Test (Non Rd. 1)	0.821	0.808	0.500	0.915	0.667	0.842	0.571

### 3.3 Model interpretation

**Figure 3:**
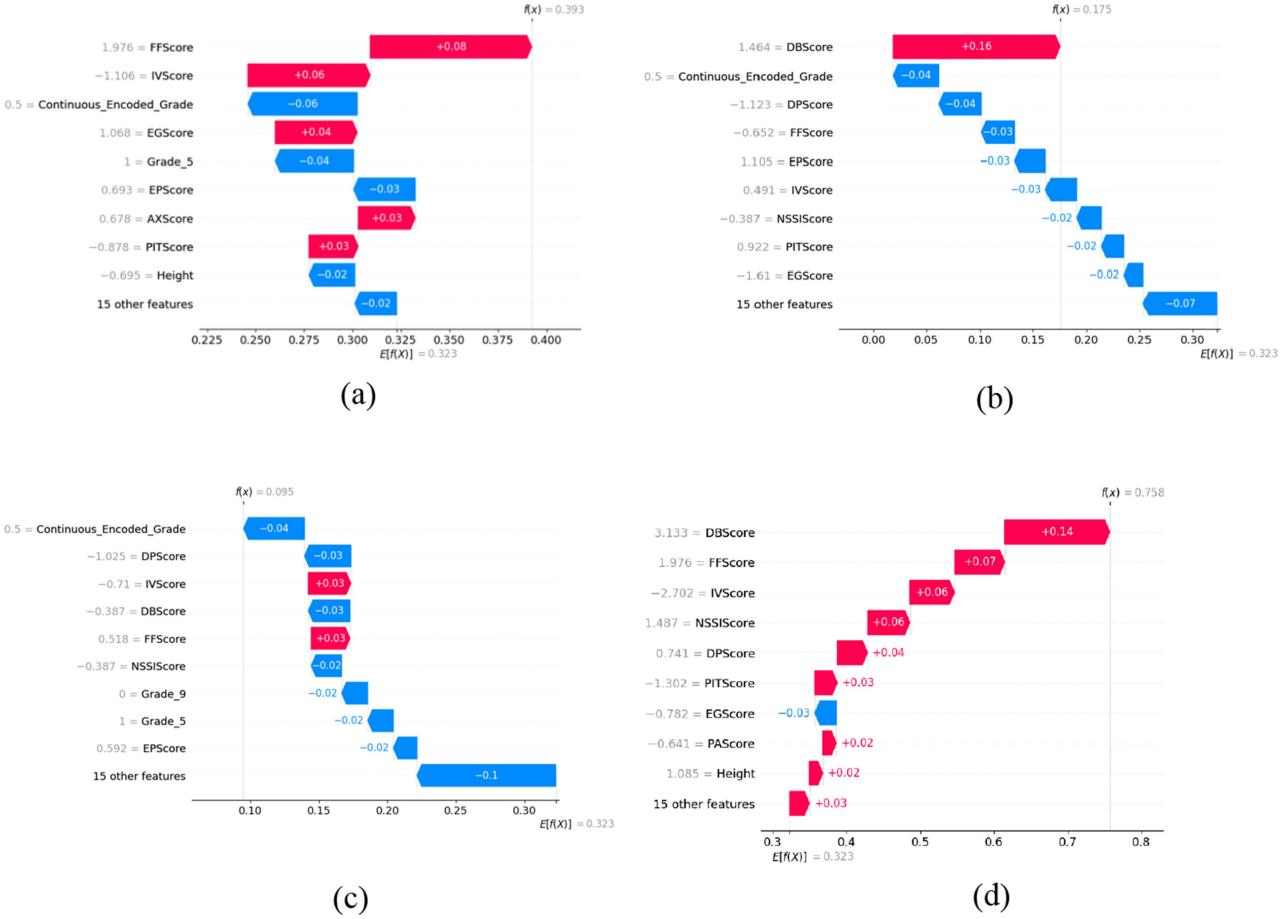
Example SHAP waterfall plots illustrating feature contributions to the ExtraRFC model’s output for different individual cases. **(a)** A typical case with moderate predicted probability. **(b)** A low-risk case with negative contributions from key features. **(c)** A very low-risk case dominated by negative feature impact. **(d)** A high-risk case with strong positive feature contributions.

**Figure 4:**
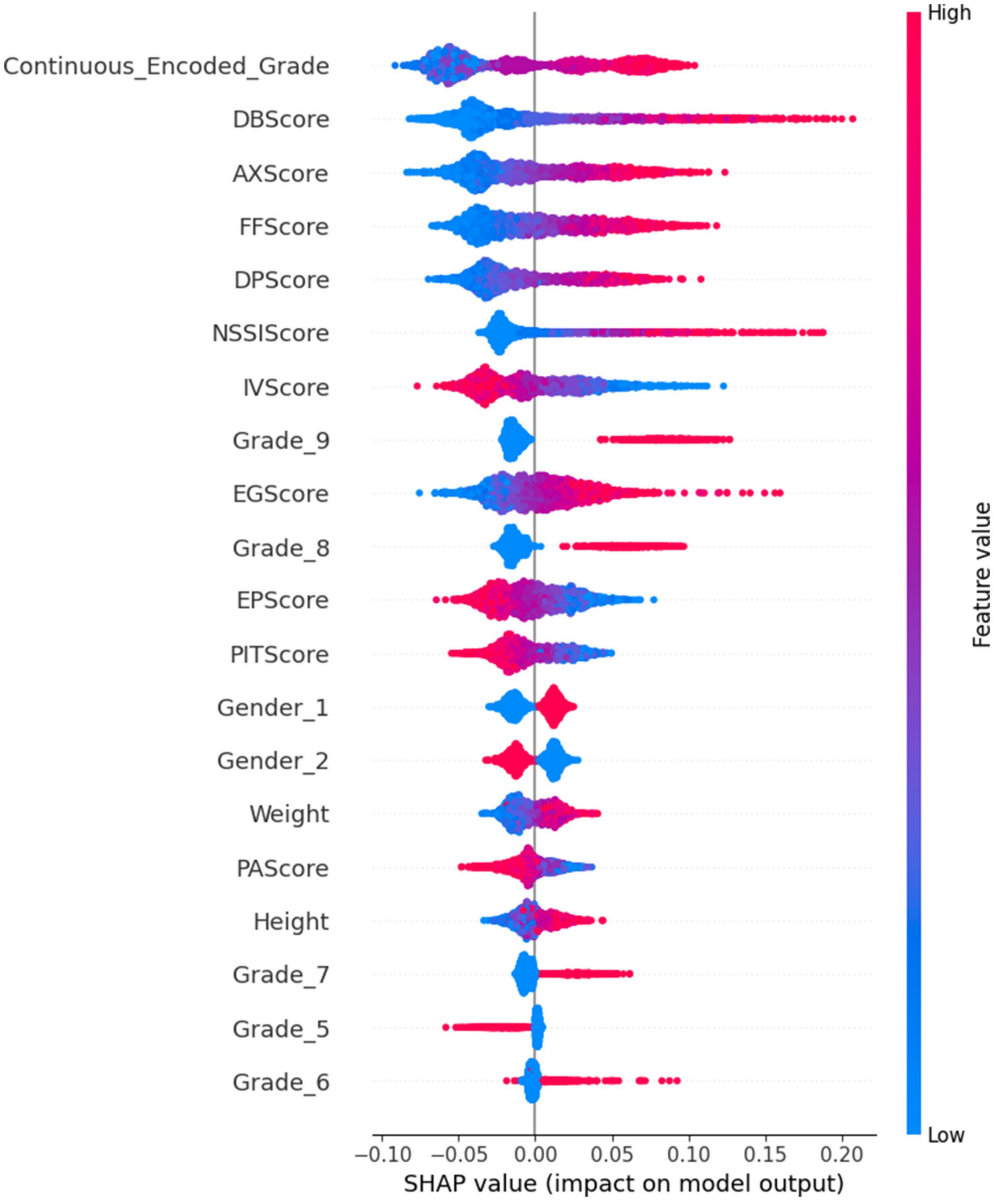
ExtraRFC SHAP Summary Plot.

The SHAP summary plot created from the test dataset on the ExtraRFC model (trained on 70% of training data - the combined data of any one-fold in the CV Training and Validation sets) was used as the tool to analyze the greatest contributing features for predicting IAT outcome in this model. Waterfall plots (Figure 3) from four test instances were also presented for further analysis. The summary plot presented in Figure 4– where blue value denotes low feature values, red denotes high feature values and purple denotes values near the mean feature value - suggests that Grade, DB Score, AX Score, FF Score and DP Score had the greatest contributions toward predictive outcomes in the ExtraRFC model, with higher values for all these features contributing to a positive prediction for instances.

The four force plots presented predicted probabilities for positive IA diagnosis at 0.393, 0.75, 0.095 and 0.758, respectively, with base value being 0.323. Figure 3d is an instance with high predicted IA diagnosis risk, with high DB Score (+0.14), FF Score (+0.07), DP Score (+0.04) pushing the prediction away from the baseline 0.323 by 0.25 toward 0.758, aligning with what was presented in the summary plot. Negative values in IV Score and PIT Score causing an increase to the predicted IA diagnosis risk also followed the summary plot, as did the negative EG Score which reduced the diagnosis risk by 0.03. Note that all features were normalized before the model was trained, so any positive or negative feature values would correspond to low or high feature values on the original scale.

## 4 Discussion

This study found that the prevalence of IA within the examined cohort (*n* = 8,824) was 33.3%, with 57.15% being male and 42.85% female. The Chi-square test revealed that all demographic factors and psycho-social factors included in this study were significantly linked to IA. This study also identified grade level, DB Score, AX Score, FF Score, and DP Score as the primary predictors of IA through ML approach. Our study demonstrated the effectiveness of the ExtraRFC model in predicting IA among children and adolescents (Grades 1 to 9) with an accuracy of 0.795 and F1 score of 0.650.

Through our analysis, we identified key predictors of IA that were consistent from previous research. The grade level stood out as the most critical factor in forecasting an individual’s IA, highlighting the significance of age. Specifically, students in Grade 8 and Grade 9 (ages 14 to 16) showed the highest percentages of IA, at 21.42 and 22.58%, respectively. This finding aligns with Karacic and Oreskovic (49), who reported the highest level of IA among the 15 to 16-year-old age subgroup.

Our findings revealed that lower AX Score and DP Score were predictive of decreased likelihood of IA, supporting Saikia et al. (50), who found significant associations between IA and psychological factors such as stress, depression, and anxiety. These associations underscored the importance of addressing emotional well-being as part of preventative strategies against IA. Additionally, our study found that lower NSSI Score is associated with a decreased likelihood of IA, suggesting that NSSI, which is linked to psychological distress such as depression, served as a predictor for IA. This supports the notion of a bidirectional relationship between IA and mental health, as discussed in studies by Lau et al. (51) and Andover et al. (52), emphasising the associations of IA and psychological well-being.

Additionally, lower FF Score, suggesting lower family dysfunction, were associated with a decreased probability of predicting IA. This was consistent with Lee & Kim (18), highlighting the role of family dynamics in IA, particularly parental satisfaction, education level, parenting style, attachment and communication within the family. This was suggests the need for fostering healthy family environments as a preventative measure against IA.

High EP and PIT Scores were associated with decreased IA likelihood, highlighting the protective role of these personal attributes. The findings also broadened our understanding of the impact of impulsiveness, relational co-dependency, gender, and age on IA, adding valuable insights to the literature (5).

Our study introduced an innovative approach by combining a prospective longitudinal methodology with machine learning to investigate IA among school children and adolescents (Grade 1 to Grade 9). The model was trained using data from wave 1, predicting IA status based on risk factors measured at the same point in time, and tested on a subsequent wave, with the goal of evaluating its ability to generalize to IA classification across different cohorts and time points. This approach allowed us to enhance the model’s generalisability by avoiding the pitfall of the model being exposed to its test data beforehand. In the process of feature selection, we employed a comprehensive approach that analyzed a sufficient range of combinations of different predictors, ultimately identifying the optimal set that delivered the best performance. Additionally, the employment of CV techniques further bolstered the robustness and reliability of our findings, allowing for a rigorous evaluation of the models against unseen data, reducing overfitting, and fine-tuning parameters. Unlike traditional statistical analyses that only pinpoint whether an association exists between certain factors and outcomes, our study employed SHAP graphs. This method indicated the direction of a risk factor’s influence on the outcome, detailing whether it contributed positively or negatively, providing more interpretable results. Consequently, it furnished more interpretable results by demonstrating not just the existence but also the magnitude, significance, and direction of associations. Hence, this approach offered both predictive power and transparency, bridging the gap between algorithmic performance and actionable understanding. Additionally, the considerable size of our dataset and the incorporation of regionally representative data, alongside the use of a validated scale, lent significant credibility and depth to our analysis.

Based on the identification of several factors closely associated with IA, we offer the following prevention recommendations for students and parents: First, improve the quality of communication and parent–child relationships within the family to reduce the risk of IA. Second, pay attention to the mental health of students, especially in reducing anxiety and depression, which can be achieved through regular mental health education and providing necessary psychological support services. Additionally, enhancing children’s empathy and positive sense of identity is also crucial for preventing IA. Finally, parents and schools should work together to educate children on how to use the internet healthily and conduct appropriate monitoring.

While our research makes significant contributions, there are several limitations. Firstly, despite the use of a large sample and regionally representative data, the research was primarily conducted in Chengdu, China, which may limit the general applicability of the findings. As a relatively developed city, the characteristics of children and adolescents (Grades 1 to 9) in Chengdu may differ from those in lesser economically developed areas, and future research needs to explore these differences. Secondly, although the schools were purposefully sampled to reflect geographic and socioeconomic diversity within Chengdu, the sample was still confined to five schools, and school type (e.g., teaching resources, student background) may introduce unmeasured confounders. Furthermore, students who graduated from the participating schools during the study period were not tracked to new schools, which may result in selective attrition and limit long-term trajectory modelling. Additionally, this study was based on self-reported data, which may introduce reporting bias. Although we used a longitudinal dataset, the present analysis focused on classifying IA status based on risk factors measured at the same time point. In other words, our ML models were developed and evaluated using data from the same wave, aiming to identify variables associated with the current presence of IA. Nevertheless, we believe there is substantial potential in further leveraging this longitudinal data. Future studies may implement trajectory prediction models, using earlier wave measures to predict IA outcomes in subsequent waves. This would allow for a more rigorous investigation into the developmental course of IA and enhance our ability to identify individuals at risk before problematic usage emerges.

## 5 Conclusion

In conclusion, this study leveraged machine learning techniques to predict IA in children, employing a longitudinal Chinese children cohort dataset. Our findings underscore the superiority of the ML model ExtraRFC in accurately identifying IA, with DB Score, AX Score, and FF Score emerging as significant predictors. This not only confirms the potential of ML in diagnosing IA but also highlights the importance of considering a multitude of factors, including demographic and psychological elements, in understanding and combating IA among children. Our research paves the way for future investigations to further refine these predictive models, offering a promising avenue for early detection and intervention strategies in the digital well-being domain.

## Data Availability

The original contributions presented in the study are included in the article/Supplementary material, further inquiries can be directed to the corresponding author/s.

## Glossary

IA: Internet Addiction
IAT: Internet Addiction Test
ML: Machine Learning
CPCD: Chengdu Positive Child Development
CBC: Cognitive Behavioral Competencies
PA: Prosocial Attributes
PI: Positive Identity
GPYDQ: General Positive Youth Development Qualities
LS: Life Satisfaction
DB: Delinquent Behavior
NSSI: Non-Suicidal Self-Injury
DP: Depression
AX: Anxiety
FF: Family Function Disorders
EG: Egocentrism
EP: Empathy
IV: Academic Intrinsic Value
UV: Academic Utility Value
SHAP: SHapley Additive exPlanations
ExtraRFC: Extra Random Forest Classifier
BernoulliNB: Bernoulli Naïve Bayes
LogisticReg: Logistic Regression
XGB: eXtreme Gradient Boosting Classifier
MLP: Multiple Layer Perceptron
Transformer: Transformer Encoder Classifier
CV: Cross-Validation
AUC-ROC: Area Under Curve - Receiver Operator Curve
AP: Average Precision (sometimes used in the context of precision-recall curves)
DCA: Decision Curve Analysis
TP: True Positives
TN: True Negatives
FP: False Positives
FN: False Negatives
NPV: Negative Predictive Value
