# Supplementary material for: Explainable machine learning prediction of internet addiction among Chinese primary and middle school children and adolescents: a longitudinal study based on positive youth development data (2019–2022)
Source: Front Public Health. 2025 Jul 16;13:1590689. doi: 10.3389/fpubh.2025.1590689 (PMC12307306; doi:10.3389/fpubh.2025.1590689)
Supplement: Supplementary file 1 [file Table_1.docx]

**Appendix Tables**

**Table A1:** Explanation of Features

| **Feature** | **Explanation** |
| --- | --- |
| Gender | Biological sex of the participant (e.g., male, female) |
| Age | The age of the participant |
| Grade | Educational level or class grade of the participant |
| Weight | The body weight of the participant, potentially in kilograms or pounds |
| Height | The height of the participant, possibly measured in centimetres or inches |
| CBCScore | Cognitive-Behavioural Competencies |
| PAScore | Prosocial Attributes Score, assessing tendencies towards helping behaviour, empathy, and cooperation |
| PITScore | Positive Identity Score, measuring the strength and positivity of the participant’s sense of self and identity |
| GPYDQScore | Score from a questionnaire measuring General Positive Youth Development Qualities |
| LSScore | Life Satisfaction Score, assessing the participant’s overall satisfaction with their life |
| DBScore | Delinquent Behaviour Score, measuring the frequency or severity of delinquent or anti-social behaviours |
| NSSIScore | Non-Suicidal Self-Injury Score, indicating the occurrence of self-harm behaviours without suicidal intent |
| DPScore | Depression Score, measuring symptoms or severity of depression |
| AXScore | Anxiety Score, assessing levels of anxiety |
| FFScore | Family Function Score, evaluating the health and functionality of family dynamics |
| EGScore | Egocentrism Score, measuring the degree to which an individual is self-centred |
| EPScore | Empathy Score, assessing the ability to understand and share the feelings of others |
| IVScore | Academic intrinsic value score, reflecting how internally motivated to learn |
| UVScore | Academic utility value score, reflecting how much the student values the task in terms of usefulness |
| IATOutcomes | Internet Addiction Test Outcomes, results or classification derived from an assessment of Internet Addiction |

**Table A2:** Optimised Hyperparameters and Features for Different Models

| Model | Hyperparameter | Value |
| --- | --- | --- |
| ExtraRFC | n_estimators max_depth max_samples | 400  48  0.55 |
|  | max_features | 0.4 |
|  | ccp_alpha | 1e-08 |
|  | features | {‘DBScore’, ‘Grade’ (One Hot Encoded), Grade (Continuously Encoded),  ‘IVScore’, ‘DPScore’, ‘NSSIScore’, ‘Weight’, ‘EGScore’, ‘AXScore’  ‘Gender’ (One Hot Encoded), ‘PITScore’, ‘Height’, ‘EPScore’, ‘PAScore’} |
| BernoulliNB | alpha fit_prior  fit_prior | 100  True |
|  | features | {‘DBScore’, ‘Grade’ (One Hot Encoded), Grade (Continuously Encoded),  ‘IVScore’, ’DPScore’, ‘NSSIScore’, ‘Weight’, ‘FFScore’, ‘EGScore’,  ‘AXScore’, ‘Gender’ (One Hot Encoded), ‘PITScore’, ‘Height’, ‘EPScore’} |
| LogisticReg | C  tol  max_iter  panelty | 10  0.01  400  ‘l1’ |
|  | features | {‘DBScore’, ‘Grade’ (One Hot Encoded), Grade (Continuously Encoded),  ‘IVScore’, ‘DPScore’, ‘NSSIScore’, ‘Weight’, ‘FFScore’, ‘EGScore’,  ‘AXScore’, ‘Gender’ (One Hot Encoded), ’PITScore’} |
| XGBoost | n_estimators max_depth subsample colsample_bytree | 50  24  0.7  0.7 |
|  | gamma | 1e-06 |
|  | eta | 0.1 |
|  | features | {‘DBScore’, ‘Grade’ (One Hot Encoded), Grade (Continuously Encoded),  ‘IVScore’, ‘DPScore’, ‘NSSIScore’, ‘Weight’, ‘FFScore’, ‘EGScore’,  ‘AXScore’, ‘Gender’ (One Hot Encoded)} |
| MLP | hidden_dim dropout natch_size  epochs  lr  batchnorm | 64  0.15  64  15  1e-4 |
|  | grad clip | True |
|  | loss | True |
|  | features | nn.CrossEntropyLoss |
|  |  | {‘DBScore’, ‘Grade’ (One Hot Encoded), Grade (Continuously Encoded),  ‘IVScore’, ‘DPScore’, ‘NSSIScore’, ‘Weight’, ‘FFScore’, ‘EGScore’,  ‘AXScore’, ‘Gender’ (One Hot Encoded), ‘PITScore’, ‘Height’, ‘EPScore’, ‘PAScore’, ‘GPYDQScore’} |
| Transformer | hidden_dim num_transformer_layer dropout  dim_feedforward natch_size  nhead | 16  1  0.1  32  32  4 |
|  | use_cls | False |
|  | epochs | 15 |
|  | lr | 0.001 |
|  | grad_clip | True |
|  | loss | nn.CrossEntropyLoss |
|  | features | {‘DBScore’, ‘Grade’ (One Hot Encoded), Grade (Continuously Encoded)  ‘IVScore’, ‘DPScore’, ‘NSSIScore’, ‘Weight’, ‘FFScore’, ‘EGScore’,  ‘AXScore’, ‘Gender’ (One Hot Encoded), ‘PITScore’, ‘Height’, ‘EPScore’,  ‘PAScore’} |

**Table A3:** Spearman’s Correlation Coefficient

| Feature1 | Feature2 | Correlation Coefficient | P-Value | |
| --- | --- | --- | --- | --- |
| Gender | Age | 0.0068 | 0.5234 |  |
| Gender | Grade | 0.0185 | 0.0824 | |
| Gender | Weight | -0.0264 | 0.0133 | |
| Gender | Height | -0.0257 | 0.0159 | |
| Gender | NakedeyevisionLeft | -0.0602 | 0.0 | |
| Gender | NakedeyevisionRight | -0.0633 | 0.0 | |
| Gender | CBCScore | 0.0311 | 0.0035 | |
| Gender | PAScore | 0.0571 | 0.0 | |
| Gender | PITScore | -0.0134 | 0.2095 | |
| Gender | GPYDQScore | 0.0392 | 0.0002 | |
| Gender | LSScore | -0.0102 | 0.3393 | |
| Gender | DBScore | -0.0935 | 0.0 | |
| Gender | NSSIScore | 0.034 | 0.0014 | |
| Gender | DPScore | 0.0019 | 0.8586 | |
| Gender | AXScore | 0.1237 | 0.0 | |
| Gender | FFScore | -0.0393 | 0.0002 | |
| Gender | EGScore | 0.0049 | 0.6467 | |
| Gender | EPScore | 0.1217 | 0.0 | |
| Gender | IVScore | -0.0093 | 0.3811 | |
| Gender | UVScore | -0.0026 | 0.808 | |
| Gender | IATOutcomes | -0.0749 | 0.0 | |
| Age | Grade | 0.976 | 0.0 | |
| Age | Weight | 0.7499 | 0.0 | |
| Age | Height | 0.8147 | 0.0 | |
| Age | NakedeyevisionLeft | -0.2912 | 0.0 | |
| Age | NakedeyevisionRight | -0.3139 | 0.0 | |
| Age | CBCScore | -0.0842 | 0.0 | |
| Age | PAScore | -0.0518 | 0.0 | |
| Age | PITScore | -0.1853 | 0.0 | |
| Age | GPYDQScore | -0.0978 | 0.0 | |
| Age | LSScore | -0.2106 | 0.0 | |
| Age | DBScore | 0.2229 | 0.0 | |
| Age | NSSIScore | 0.0979 | 0.0 | |
| Age | DPScore | -0.003 | 0.7802 | |
| Age | AXScore | 0.0267 | 0.0121 | |
| Age | FFScore | 0.0048 | 0.6535 | |
| Age | EGScore | 0.0158 | 0.1374 | |
| Age | EPScore | -0.0723 | 0.0 | |
| Age | IVScore | -0.3702 | 0.0 | |
| Age | UVScore | -0.2777 | 0.0 | |
| Age | IATOutcomes | 0.3105 | 0.0 | |
| Grade | Weight | 0.7625 | 0.0 | |
| Grade | Height | 0.829 | 0.0 | |
| Grade | NakedeyevisionLeft | -0.2981 | 0.0 | |
| Grade | NakedeyevisionRight | -0.3236 | 0.0 | |
| Grade | CBCScore | -0.0776 | 0.0 | |
| Grade | PAScore | -0.042 | 0.0001 | |
| Grade  Grade  Grade | PITScore  GPYDQScore  LSScore | -0.1824  -0.0878  -0.2085 | 0.0  0.0  0.0 | |

**Table A4:** DeLong Test Results

| P values | ExtraRFC | BernoulliNB | LogisticReg | XGBoost | MLP | Transformer |
| --- | --- | --- | --- | --- | --- | --- |
| ExtraRFC | N/A | 4.06e-5 | 0.005 | 0.003 | 0.018 | 0.005 |
| BernoulliNB | 4.06e-5 | N/A | 0.051 | 0.152 | 0.051 | 0.144 |
| LogisticReg | 0.005 | 0.051 | N/A | 0.933 | 0.597 | 0.995 |
| XGBoost | 0.003 | 0.152 | 0.933 | N/A | 0.587 | 0.932 |
| MLP | 0.018 | 0.051 | 0.680 | 0.597 | N/A | 0.594 |
| Transformer | 0.005 | 0.014 | 0.995 | 0.932 | 0.594 | N/A |

**Appendix Figures**

**
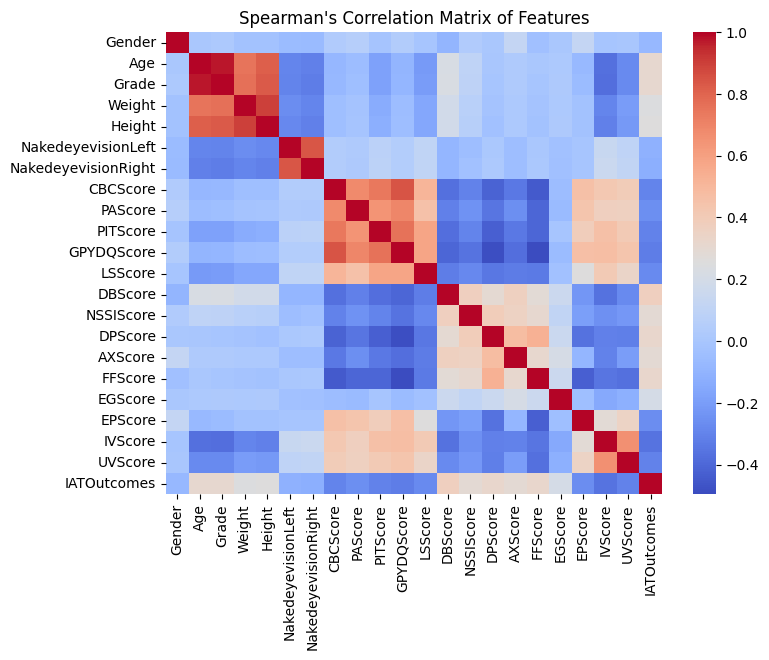
**

**Fig. A1:** The Heatmap of Spearman Correlation Analysis Between Variables

| **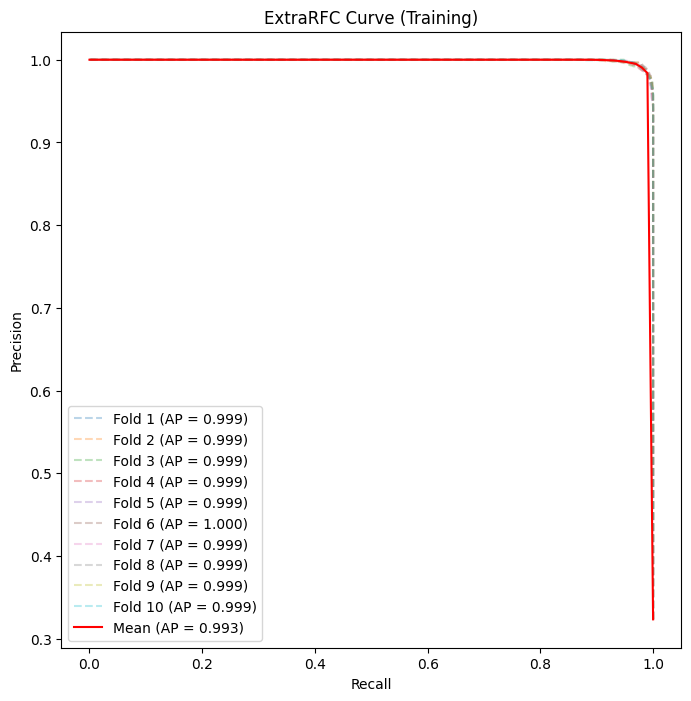**(a) ExtraRFC 10CV Training PR Curves | **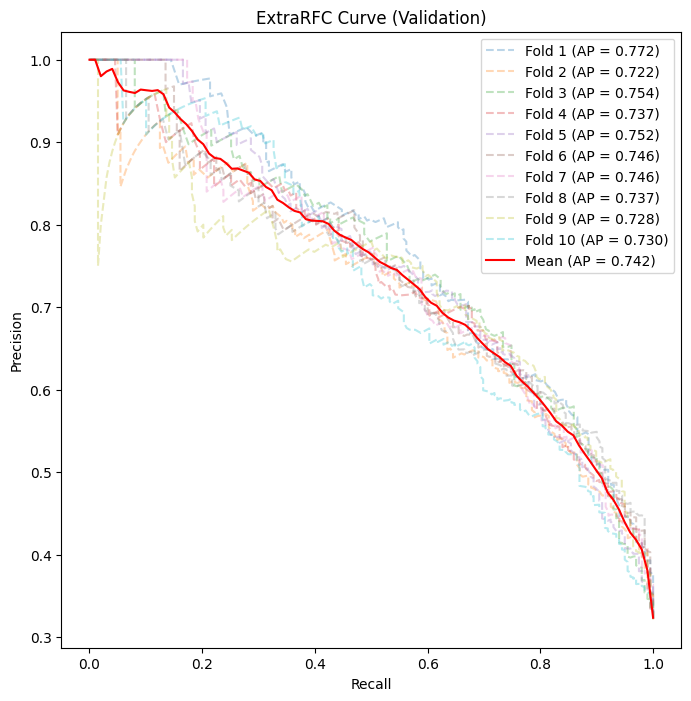**(b) ExtraRFC 10CV Validation PR Curves |
| --- | --- |

**Fig. A2:** ExtraRFC PR Curves

| **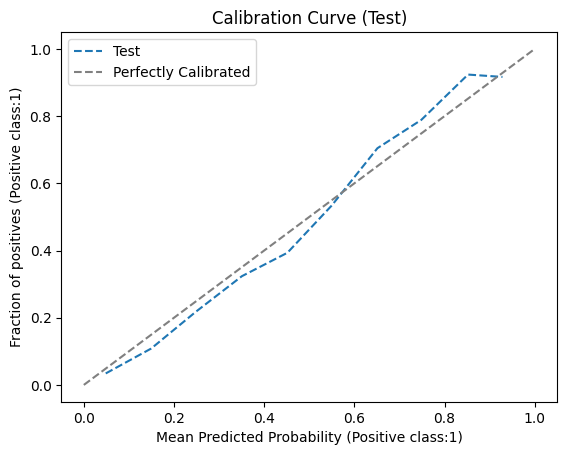**(a) ExtraRFC Test Calibration Curves | **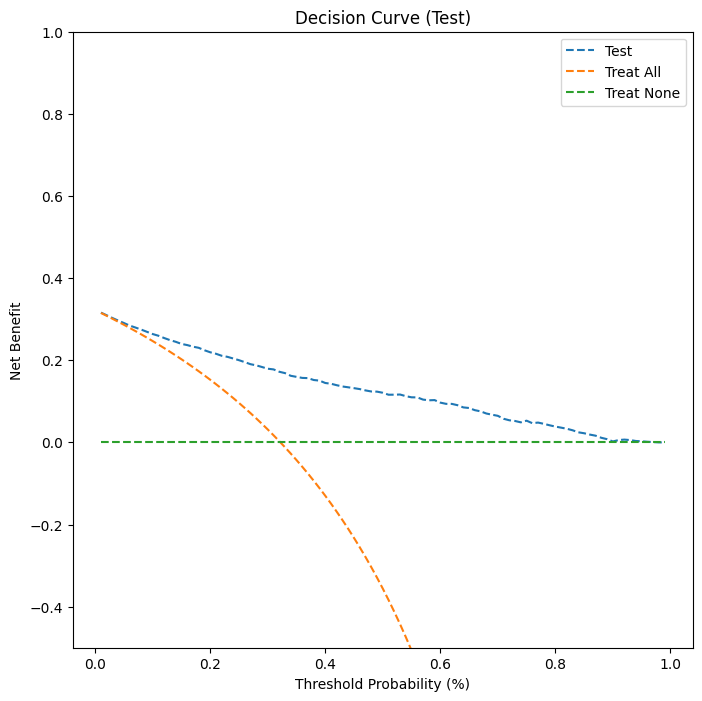**  (b) ExtraRFC Test Decision Curve |
| --- | --- |

**Fig. A3:** ExtraRFC Test Calibration and Decision Curves

**
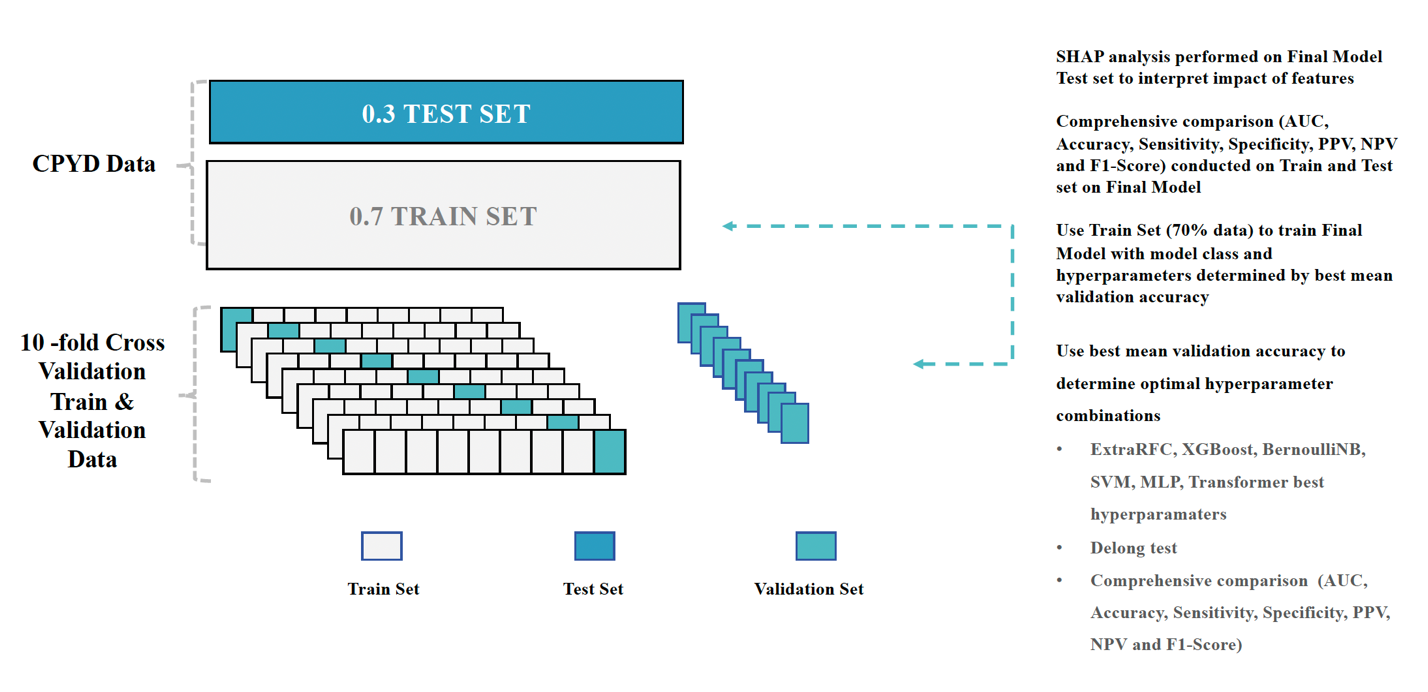
**

**Fig. A4:** Experimental Framework Data Usage Diagram
